# 

**Published:** 1895-03-23

**Authors:** 


PRICE TWOPENCE.

No. 443, Vol. XVII,—March 23rd, 1895.

[Registered at the General Post Office as a Newspaper.

WITH EXTRA NURSING SUPPLEMENT.

Per Annum, 10s. 6d., post free.

Monthly Parts, 6d.; post free, 8d.

Ditto per Annum, 8s. 6d., post free.

### THE HOSPITAL

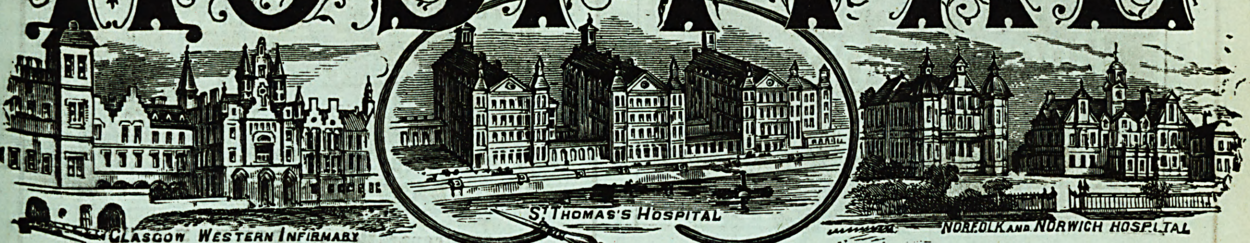

No. 443. Vol. XVII. WITH EXTRA NURSING SUPPLEMENT. SATURDAY, MARCH 23RD, 1895.

